# Regulation of T-independent B-cell responses by microRNA-146a

**DOI:** 10.3389/fimmu.2022.984302

**Published:** 2022-09-12

**Authors:** Jennifer K. King, Tiffany M. Tran, May H. Paing, Yuxin Yin, Amit K. Jaiswal, Ching-Hsuan Tso, Koushik Roy, David Casero, Dinesh S. Rao

**Affiliations:** ^1^ Department of Medicine, David Geffen School of Medicine, University of California Los Angeles, CA, United States; ^2^ Department of Pathology & Laboratory Medicine, David Geffen School of Medicine, University of California Los Angeles, CA, United States; ^3^ Department of Pathology, University of Utah Salt Lake City, UT, United States; ^4^ F Widjaja Foundation Inflammatory Bowel and Immunobiology Research Institute, Cedars-Sinai Medical Center, Los Angeles, CA, United States; ^5^ Jonsson Comprehensive Cancer Center, University of California Los Angeles, CA, United States; ^6^ Eli and Edythe Broad Center of Regenerative Medicine and Stem Cell Research, University of California Los Angeles, CA, United States

**Keywords:** B-cell, microRNA, extrafollicular B cell response, Traf6, NFkB

## Abstract

The microRNA, miR-146a, is a negative feedback regulator of the central immune transcription factor, nuclear factor kappa B (NFkB). MiR-146a plays important roles in the immune system, and miR-146a deficient mice show a complex phenotype with features of chronic inflammation and autoimmune disease. In this study, we examined the role of miR-146a in extrafollicular B-cell responses, finding that miR-146a suppresses cellular responses *in vivo* and *in vitro*. Gene expression profiling revealed that miR-146a-deficient B-cells showed upregulation of interferon pathway genes, including *Traf6*, a known miR-146a target. We next interrogated the role of TRAF6 in these B-cell responses, finding that TRAF6 is required for proliferation by genetic and pharmacologic inhibition. Together, our findings demonstrate a novel role for miR-146a and TRAF6 in the extrafollicular B-cell responses, which have recently been tied to autoimmune disease pathogenesis. Our work highlights the pathogenetic role of miR-146a and the potential of pharmacologic inhibition of TRAF6 in autoimmune diseases in which miR-146a is deregulated.

## Introduction

MicroRNAs (miRNAs) are post-transcriptional regulators of gene expression, acting *via* the RNA-induced silencing complex to repress a range of mRNA targets. The miRNA, miR-146a, is widely expressed throughout the immune system, and is induced upon immune cell activation by the pleiotropic transcription factor NFkB. The targets of miR-146a include a range of mRNA molecules that are critical in immune cell activation, such as *Traf6* and *Irak1*, whose downregulation contributes to immune system homeostasis by terminating activation (1). Hence, miR-146a is a feedback regulator of the NFkB pathway. This critical function of miR-146a is highlighted by the finding of chronic inflammation and autoimmune disease in *Mir146a*-/- (miR-146aKO) mice, in which the loss of feedback inhibition led to chronic NFkB activity. Since the role of miRs may vary within different tissues with discrete molecular targets, it is important to study their roles in cell-specific contexts. Elegant studies have elucidated miR-146a’s cellular and molecular roles in myeloid cells (2, 3), T-cell subsets (4–7), NK cells (8), and germinal center (GC) B-cell responses (4, 5, 9, 10). Interestingly, the role of miR-146a in regulating germinal center B-cell responses are dependent on T follicular helper cells *via* the ICOS-ICOSL and the CD40 signaling pathways, amongst others. However, the role of miR-146a in B-cells in the context of T-independent responses has yet to be more fully described.

T-cell independent B-cell responses have been studied extensively in recent years as fundamentally important process in immunity, and, increasingly, as potentially important mechanisms in autoimmune disease. During infection, bacteria or viruses activate immune cells *via* toll-like receptors (TLRs) and other pattern recognition receptors on antigen presenting cells, including B-cells. Supporting this idea, previous studies have shown significant polyreactivity of naïve B-cells to TLR ligand stimulation (i.e. ssDNA, lipopolysaccharide) (11). B-cell stimulation with ligands engaging TLR4, 7, and 9 underlies responses including proliferation, differentiation, and class switch recombination (12). Engagement of TLRs results in activation of a pro-inflammatory signaling cascade that involves TRAF6, IRAK1, and MyD88, which eventually activates NFkB and mitogen-activated protein kinase pathways (13). In the course of normal immune responses and in autoimmunity, such activation and subsequent cytokine production may lead to further innate and adaptive responses. Hence, the regulation of these responses, particularly the appropriate termination of such responses, is critically important in maintaining overall heath and preventing immune responses that are deleterious to the individual.

Given the role of miR-146a in negative feedback regulation of immune responses, we studied T-independent B-cell responses using the miR-146aKO model (2). First, we phenotypically characterized miR-146a regulation of B-cell function both *in vivo* and *in vitro*, finding that B-cell responses to T-independent stimuli were heightened in miR-146aKO mice. We then characterized molecular mechanisms in miR-146a-dependent B-cell activation, identifying enrichment in the IFNγ pathway, and *Traf6* as an important miR-146a regulatory target. Modulating *Traf6* levels *in vivo*, we found a graded phenotypic reversal of exaggerated T-independent B-cell responses in miR-146aKO. Targeting TRAF6 pharmacologically, we found that miR-146aKO B-cells showed a higher sensitivity to TRAF6 inhibition by a small molecule inhibitor. Together, our studies suggest that miR-146a plays a direct role in regulating B-cell immune responses to T-independent stimuli. Furthermore, inhibition of TRAF6 by genetic and pharmacologic means is key to inhibiting these B-cell responses, and potentially, in treating disease conditions where miR-146a is deregulated.

## Materials and methods

### Mice

C57B/6 wild-type (WT) and Cas9-eGFP (14) mice were purchased from Jackson Laboratories. miR-146a-deficient (miR-146aKO) mice were created as previously described (2) in C57B/6 stock background. Females were used due to their higher predisposition for lupus-like autoimmunity features as described (2). miR-146aKO mice were bred onto Cas9-eGFP mice to generate miR-146aKO-Cas9-eGFP mice (M146aKOCas). All animal studies were approved by the UCLA Office of Animal Research Oversight.

### *In vivo* immunization

MiR-146aKO and WT mice (<24 weeks) were immunized by intraperitoneal injection with of TNP-LPS (Biosearch Technologies) in PBS (25ug in 200ul) Serum was analyzed *via* indirect ELISA at 0, 3, 7, 14, and 28 days for TNP-LPS specific antibodies and total IgM and IgG.

### B-cell isolation and *in vitro* stimulations

Spleens from mice were pooled from 3-8 mice/group, and B-cells were sorted using either CD43 negative selection as previously described (15) or B220^+^ positive selection, confirmed *via* B220+ FACS staining (>95% purity). Both methods confirmed similar *in vitro* phenotypic responses. B-cells were cultured in complete media (C10), with lipopolysaccharide (10ug/ml; Sigma) or Class B CpG ODN 1668 (1000ng/ml; InVivoGen). Cultures were harvested at 0, 24, 48, 72, 96 hours. Cell counts were confirmed both manually and *via* FACS using Calibrite beads as described (16). Marginal Zone (MZ) and Follicular (FO) B-cell Isolation Kits (Miltenyi) were used, which first depletes non-B cells, B1 cells, and transitional B cells, while then enriching for MZ and FO cells. Final sorted subsets were obtained with >95% purity on B220+ cells and confirmed *via* CD21^+^ and CD23^+^. Cell counts were calculated according to FACS confirmation. TRAF6-Ubc13 inhibitor, C25-140 (MedChemExpress) (17) in DMSO carrier, was added at various concentrations 24 hours prior to the examined time point for *in vitro* culture stimulations.

### RNA-seq

Total RNA was purified from B-cells and cDNA libraries were constructed using the Illumina TruSeq RNA Sample kit. Analysis was performed as described (18). Differentially expressed genes were compared in WT and miR-146aKO B-cells at specific time points after stimulation and analyzed *via* Geneset Enrichment Analysis (GSEA) (19). For each GSEA test, genes were pre-ranked based on observed RNA-Seq moderate fold-changes between any two conditions (e.g 72h LPS in WT *vs*. miR-146aKO). Hallmark gene sets from MSigDB (20) were employed. To visualize differential expression, heatmaps were generated using the R heatmap.2 function using average z-scores per treatment group. The data presented in the study are deposited in the NCBI Sequence Read Archive (SRA), accession number PRJNA723980.

### mRNA and miR qPCR

RNA isolation and RT-qPCR were performed as previously described (21). miR-146a expression was measured using TaqMan miR assays and a StepOne Plus Real-time PCR System, with snoRNA202 (control).

### Western blot analysis

Anti-TRAF6 (MBL) and anti-β-Actin were used, and western blots were performed as previously described (21).

### TIDE (Tracking of Indels by DEcomposition) analysis

PCR was amplified using Q5^®^ Hot Start High-Fidelity 2X Master Mix, and purified using SpinSmart™ PCR Purification & Gel Extraction Columns. Sanger sequencing was done by Laragen and analyzed using TIDE version 2.0.1 (https://tide.deskgen.com/).

### Bone marrow transplantation

5-florouracil-enriched donor bone marrow from young M146aKOCas mice was spin infected with MSCV-sgRNA vectors containing sgRNAs against *Traf6*. Bone marrow cells were injected retro-orbitally into lethally irradiated syngeneic recipient mice (8 per group) for reconstitution. At 3 months, bone marrow and spleens were harvested, pooled, and sorted for sample collection or *in vitro* B cell cultures. Engraftment of donor cells (GFP^+^) was at least >95%.

### Statistical analysis

Two-tailed Student’s *t* test or an ordinary one-way ANOVA followed by Tukey’s multiple comparisons test were performed. Data are presented as the mean ± SEM, unless otherwise indicated. *P* < 0.05 was denoted as significant. *P < 0.05, **P < 0.01, ***P < 0.001, and ****P < 0.0001. NS: not significant.

## Results

### miR-146aKO mice demonstrate heightened responses to *in vivo* immunization with t-independent antigen

**Figure 1:**
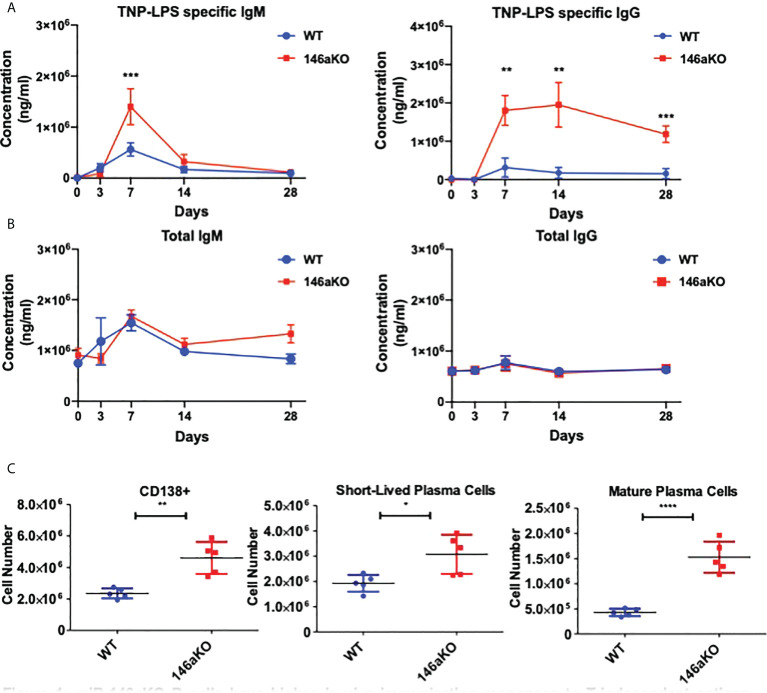
miR-146aKO B cells have higher *in vivo* immunization responses to T-independent antigen than WT. **(A)** Primary immunization antibody response to T-independent antigen at day 28 show TNP-LPS specific antibodies *vs*. **(B)** total IgM, IgG antibodies. **(C)** FACS analysis of plasma cells and subsets at endpoint. n = 6-9 mice/group. All analysis used *t* test for respective time point comparisons; *P < 0.05, **P < 0.01, ***P < 0.001, ****P < 0.0001. Data represent mean ± SEM.

To first assess the effects of T-independent antigen on an organismal level, we investigated primary immunization B-cell responses *in vivo*. We immunized WT and miR-146aKO mice with the T-cell independent antigen, TNP-LPS, and collected serum to measure primary antibody responses. MiR-146aKO serum had significantly higher levels of both TNP-LPS specific IgM at day 7, and TNP-LPS specific IgG at day 7, 14, and 28 (**Figure 1A**). While production of IgM is traditionally thought to predominate during T-independent antigen immune responses, it is now well-known that IgGs may also be produced (22). Overall total IgM and total IgG showed no statistically significant differences in KO *vs*. WT primary immunization responses **(** **Figure 1B** **)**. At day 28, we examined splenic plasma cell (PC) subsets. An increased number of total CD138+ PCs were seen in the miR-146aKO spleens **(** **Figure 1C** **).** We further characterized PCs and found that short-lived plasma cells/plasmablasts (CD138^+^B220^+/lo^) and mature PCs (CD138^+^B220^-^) **(** **Supplement 1** **)** were both significantly higher in miR-146aKO spleens (**Figure 1C**). Such differences were not seen in the bone marrow, although such studies were limited due to the complex hematopoietic phenotype that occurs in aging miR-146aKO mice as previously described (23). Also, we independently confirmed that immunization with a T-dependent antigen resulted in higher peptide-specific IgG secretion in miR-146aKOs **(** **Supplement 2A** **).** Taken together, these data indicate that miR-146aKO mice have a stronger response to *in vivo* immunization with T-independent antigen, in addition to the previously described augmented response to T-dependent antigens.

### miR-146aKO B-cells have greater response to TLR ligands

**Figure 2:**
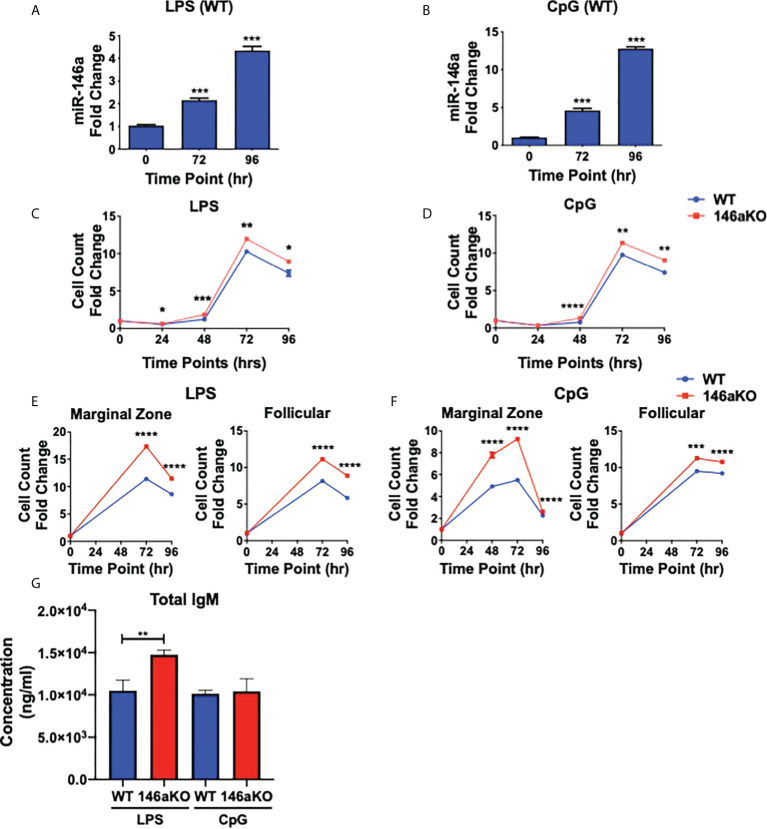
miR-146aKO B cells have higher *in vitro* response to T-independent antigens than WT and demonstrate increased antibody secretion in response to stimulation. **(A, B)** Stimulation of WT native splenic B-cells with **(A)** LPS or **(B)** CpG shows significant miR-146a induction at time points 72 and 96 hr (one-way ANOVA with Tukey’s multiple-comparisons test; ***P < 0.001). Graph combines three independent experiments. n = 3-4 mice/group/exp. Data represent mean ± SEM. (C and D) miR-146aKO splenic B-cells stimulated with **(C)** LPS or **(D)** CpG have higher cell counts than WT at various time points (*t* test; *P < 0.05, **P < 0.01, ***P < 0.001, ****P < 0.0001). Representative of 3-4 mice/group in triplicates, confirmed in at least six independent experiments. **(E, F)** miR-146aKO marginal zone and follicular B-cells show higher cell count than WT when stimulated with **(E)** LPS or **(F)** CpG (*t* test; ***P < 0.001, ****P < 0.0001). Representative of three mice/group in triplicates, confirmed in three independent experiments. **(G)** Supernatants from *in vitro* stimulation at 72hr show higher total IgM secretion in miR-146aKO B cells compared to WT.

Previous studies have shown that miR-146a is highly induced in WT myeloid cells and dendritic cells stimulated with LPS (TLR4 agonist) or CpG (TLR9 agonist) (2, 3), and less so with other TLR agonists such as poly(I:C) (TLR3 agonist) and imiquimod (TLR7 agonist) (3). Therefore, to next assess the importance of miR-146a induction in B-cells, splenic B220^+^ cells from WT mice were stimulated with LPS or CpG (**Figures 2A, B** **)**. MiR-146a expression in WT was strongly induced by stimulation at 72 and 96 hours. Then, to assess the effect of miR-146a on B-cell activation, we examined *in vitro* responses among WT and miR-146aKO B-cells in response to LPS and CpG. At various time points, both LPS and CpG stimulated B-cells showed a significantly higher number of cells in miR-146aKO than in WT (**Figures 2C, D** **)**. To examine miR-146a’s effect on mature B-cell subsets, MACS-sorted marginal zone (MZ) and follicular (FO) B-cells were stimulated with LPS and CpG. At 72 and 96 hours, there was a significantly higher number of MZ B-cells and, to a lesser extent, increased FO B-cells in miR-146aKO than in WT (**Figures 2E, F** **)**. This is consistent with studies that MZ B-cells are more highly responsive to T-independent stimulation than FO B-cells (24, 25). Together, these results indicate that miR-146a-deficient B-cells stimulated with TLR ligands have higher responses than WT. Of note, we also independently verified that *in vitro* B-cell stimulation with conditions replicating T-dependent activation, CD40 +IL-4, also produced higher cell counts in miR-146aKO *vs*. WT **(** **Supplement 2B** **)**, which is consistent with previous studies assessing miR-146a’s importance in T-dependent GC responses (9). In addition to higher cell counts, *in vitro* culture supernatants for LPS miR-146aKO showed statistically significant higher amounts of total IgM compared to WT (**Figure 2G**). Thus, miR-146a deficiency results in higher *in vitro* responses in T-independent activation.

### miR-146aKO B-cells have higher activation markers to TLR s

**Figure 3:**
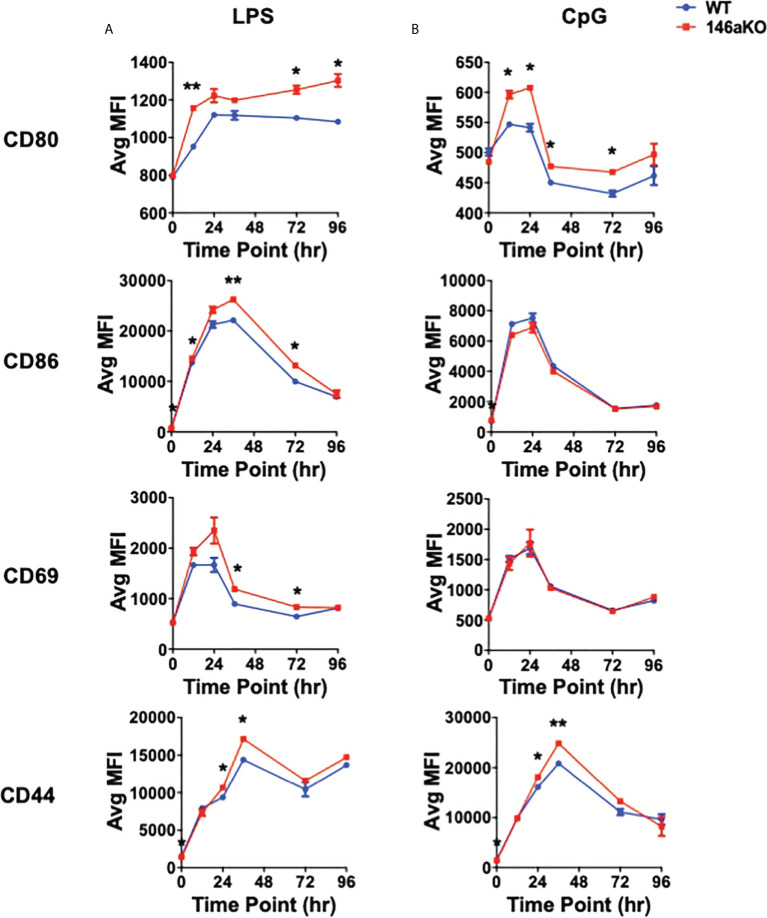
miR-146aKO B-cells have higher *in vitro* activation to T-independent antigens than WT. **(A, B)** FACS analysis of B-cell surface activation and/or co-stimulation markers are higher in miR-146aKO *vs*. WT when stimulated with **(A)** LPS and **(B)** CpG (*t* test for respective time point comparisons; *P < 0.05, **P < 0.01). Representative of three mice/group in duplicates, repeated in two independent experiments.

To further understand cellular changes between WT and miR-146aKO, we analyzed B-cell surface markers, which have been shown to respond to TLR activation, including co-stimulatory markers CD80 (B7-1) and CD86 (B7-2), early lymphocyte activation marker CD69, and activated surface glycoprotein CD44 (26). With LPS, miR146aKO B-cells had a higher median fluorescence intensity (MFI) of CD80, CD86, CD69, and CD44 than WT B-cells (**Figure 3A** **;** **Supplement 3**). MiR-146aKO B-cells also showed higher MFI of CD80 and CD44 with CpG (**Figure 3B**), although these were generally less robust compared with LPS. Hence, miR-146aKO B-cells have higher surface activation markers than WT at various time points, highlighting the idea that miR-146a deficiency leads to exaggerated phenotypic responses in B cells to TLR ligands.

### miR-146aKO B-cells have deregulated interferon pathway gene expression

**Figure 4:**
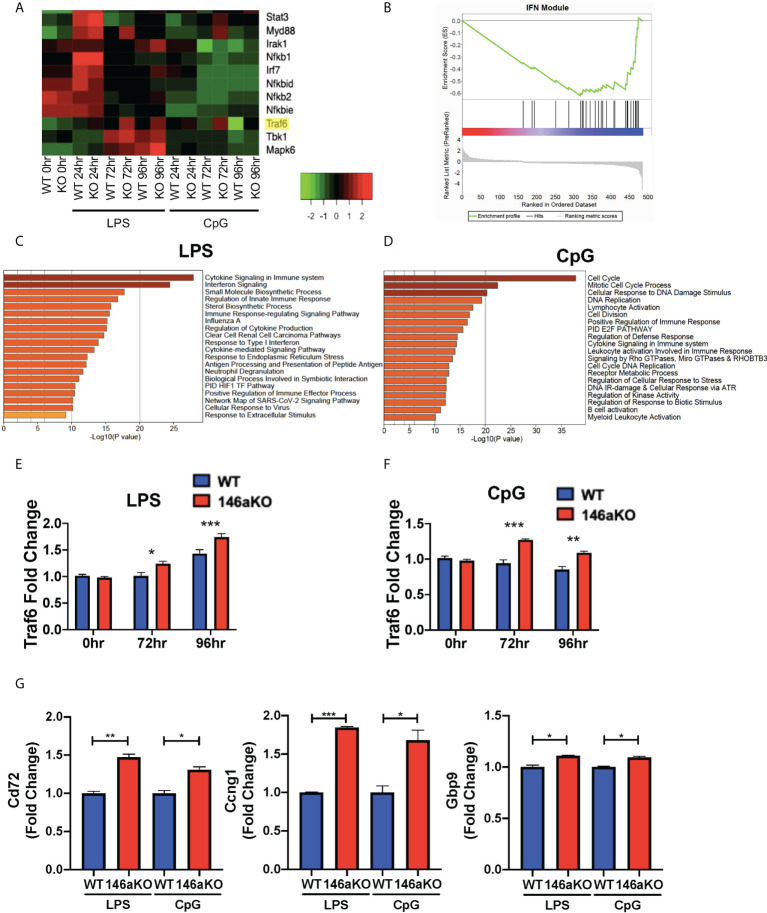
miR-146aKO B-cells have upregulated interferon pathway genes expression and *Traf6* is a target for miR-146a dependent activation. **(A)** Snapshot of heatmap from the RNA-Seq data showing differentially expressed IFN targeted genes. miR-146a molecular target, *Traf6*, is highlighted in yellow. **(B)** GSEA of genes differentially expressed between WT and miR-146aKO activated B-cells from LPS stimulation at 96 hours shows enrichment in Interferon Modules within SLE Disease. Normalized Enrichment Score: -1.94; FDR q value: 0.003. **(C)** Metascape enrichment analysis of differentially expressed genes between WT and miR-146aKO B-cells with LPS and **(D)** CpG stimulation show highest enrichment for genes within the Reactome Cytokine Signaling in Immune System and Interferon Signaling Pathways. **(E)** RT-qPCR validation for *Traf6* expression with LPS and **(F)** CpG stimulation. One-way ANOVA with Tukey’s multiple-comparisons test; *P < 0.05, **P < 0.01, ***P < 0.001. **(G)** RT-qPCR of downstream Traf6 genes in LPS and CpG-stimulated B-cells. t test; *P < 0.05, **P < 0.01, ***P < 0.001. Data represent mean ± SEM. Graphs combine three independent experiments (n = 3-4 mice/group/exp).

To study the molecular mechanisms underlying the heightened B-cell responses in miR-146aKO mice, we performed RNA-Seq analysis on splenic WT and miR-146aKO B-cells post-stimulation with LPS or CpG *in vitro* (as above) (**Figure 4A**; **Supplement 4A**). Utilizing Metascape enrichment analysis (27), we determined significant enrichment for the Cytokine Signaling in Immune System Reactome pathway for significantly differentially expressed genes post-stimulation with LPS and CpG and the Interferon Signaling Reactome Pathway with LPS (**Figures 4C** **, D**). In line with this, GSEA of differentially expressed genes at different stimulation times showed significant enrichment in interferon γ (IFN) response genes (**Supplement 4B**), which have been shown to be produced by B-cells, especially when stimulated with TLR ligands such as LPS and CpG (28, 29). Interestingly, we also found significant enrichment in IFN-annotated modules using GSEA analysis, which are known to be specifically associated with autoimmune diseases such as lupus (SLE) and rheumatoid arthritis (**Figure 4B**) (30, 31).

Given the importance of IFN pathways in autoimmune disease pathogenesis, we sought to elucidate the specific genes that were enriched. We performed unsupervised hierarchical clustering of IFN pathway gene expression across the RNA-Seq dataset (32, 33). We found several distinct clusters of genes, with the majority showing concordant responses to stimulation (LPS or CpG) between mice. However, a small subset of genes showed a distinctive pattern of expression, with the most significant changes seen between WT and miR-146aKO mice following stimulation, such as *Traf6*, *Tbk1*, and *Mapk6* (**Figure 4A** **;** **Supplement 4A**). Of these, *Traf6* is well described as a miR-146a target in macrophages and T-cells (2, 3, 7). In addition, miR-146aKO mice with attenuated *Traf6* gene expression (*miR-146a^-/-^Traf6 ^+/-^* mice) showed rescue of autoimmune phenotypes (34). Based on our RNA-Seq data (**Figure 4A**; **Supplement 4A** **)** and RT-qPCR confirmation (**Figures 4E** **, F** **)**, *Traf6* expression levels showed subtle, but significant increases in miR-146aKO splenic B-cells following both LPS and CpG stimulation. Subsequent analysis identified similarly higher protein expression of TRAF6 in the miR-146aKO spleen B-cells (**Supplement 4C, D**). Other studies have independently confirmed higher protein expression of TRAF6 in miR-146a deficient B-cells and splenocytes (2, 9, 34). To define the relative contribution of TRAF6 deregulation, we compared our dataset to a well-characterized, publicly available dataset of differentially expressed genes in *Traf6*-deficient mice (35), finding a significant overlap between our datasets (hypergeometric test: 8.39852x10^-09^). Downstream *Traf6* genes, such as *Cd72*, *Ccng1*, and *Gbp9*, which were downregulated with *Traf6* deficiency, were significantly increased in miR-146aKO splenic B-cells with LPS and CpG stimulation (**Figure 4G**). Hence, we show that miR-146aKO B- cell expression of *Traf6* RNA and protein expression increase with T-independent stimuli, resulting in a concomitant increase in *Traf6* downstream regulated genes likely contributing to and/or resulting from deregulation of IFNγ-related pathways.

### CRISPR/Cas9-induced depletion of *Traf6* in miR-146aKO results in decreased miR-146a KO B-cell response

**Figure 5:**
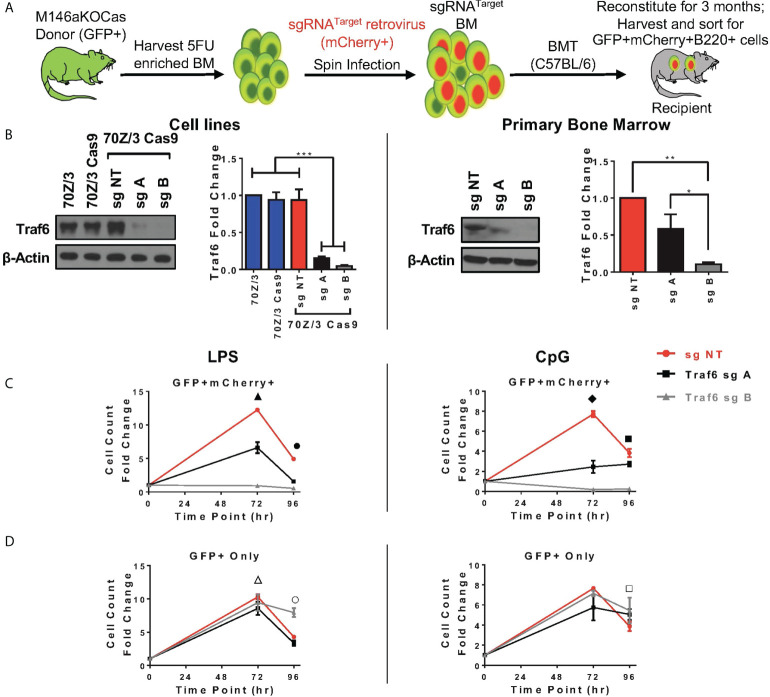
*In vivo Traf6 r*eduction results in graded decrease of miR-146a mediated B-cell response. **(A)** Schema of BMT for *Traf6* guides. **(B)** TRAF6 expression *via* Western Blot. Left panel: validation of TRAF6 deletion in 70Z/3 Cas9 cells. Repeated in duplicate. Right panel: Sorted reconstituted recipient GFP+ mCherry+ bone marrow cells show TRAF6 deletion levels. Data represent three independent experiments. One-way ANOVA with Tukey’s multiple-comparisons test; *P < 0.05, **P < 0.01, ***P < 0.001. **(C, D)** Splenic B-cells from BMT recipients are stimulated *ex vivo* with LPS (left) or CpG (right). **(C)** M146aKOCas donor cells (GFP+) with sg transduced (mCherry+) cells show NT control (red) with high cellular response (similar to native miR-146aKO stimulations in **Figures 2C, D**), but with graded decreased response in cells with *Traf6* deletion (black and gray). **(D)** M146aKOCas donor (GFP+) cells without sg RNA transduction look similar among groups (red, black, gray). All bone marrow recipient groups: n = 8 mice/group, total 24 mice/exp; repeated in two independent experiments. *t* test; *P < 0.05, **P < 0.01, ***P < 0.001. All data represent mean ± SEM. M146aKOCas, MiR-146aKO-Cas9-eGFP mice; BMT, Bone Marrow Transplantation. ▲: *** NT vs. A, **** NT vs. B, *** A vs. B; ●: **** NT vs. A, **** NT vs. B, *** A vs. B; ◆: ***NT vs. A, **** NT vs. B, ** A vs. B; ■ : * NT vs. A, *** NT vs. B, **** A vs. B; △: * NT vs. A; ○ : * NT vs. A, *** NT vs. B, *** A vs. B; □ : * NT vs. A.

Here, we sought to understand if modulation of *Traf6* in miR-146aKO cells would reverse the increased cellular responses that we had observed. We first designed multiple single guide RNAs (sg) targeting *Traf6*, and ultimately 2 guides (sgA, sgB) and one non-targeting control (sgNT) were selected after testing (**Supplement 5A**). SgRNAs were then cloned into MSCV-based retroviral vector containing RNA polymerase III U6 promoter driving expression of sgRNAs and scaffold sequence, with mCherry reporter cassette driven under a separate RNA polymerase II promoter to mark transduction, which we recently described (36). TRAF6 protein depletion was validated in a murine pre-B cell line, 70Z/3 (**Figure 5B**, left). Next, Cas9-eGFP mice were bred onto miR-146aKO mice to create miR-146aKO-Cas9-eGFP (M146aKOCas). Donor bone marrow cells from young M146aKOCas mice were spin infected with MSCV-sgRNA vectors and injected into irradiated recipients (18). Following 3 months of reconstitution, bone marrow and spleen cells were sorted for *ex vivo* B-cell cultures as in **Figure 1** (**Figure 5A**).

For verification of CRISPR editing, we sorted GFP^+^mCherry^+^ primary bone marrow cells for Sanger sequencing and tracking of indels by decomposition (TIDE) (**Supplement 5B**). As predicted, sequencing showed predicted disruptions of the target gene (**Supplement 5B, left**). Interestingly, TIDE analysis showed total efficiency for sgA as 49.4%, and sgB as 73.6%, suggesting greater gene editing efficiency using sgB than sgA (**Supplement 5B, right**). This is consistent with the finding that various guides express different efficiencies for gene editing (37). Protein TRAF6 deletion was confirmed using sorted GFP^+^mCherry^+^ primary bone marrow cells from transplanted mice (**Figure 5B**, right). Of note, lower protein expression of TRAF6 was seen in both the 70Z/3 and bone marrow, with the following pattern: sgNT > sgA > sgB. Hence, we demonstrate successful CRISPR/Cas9-induced *in vivo* depletion of *Traf6* in reconstituted miR-146aKO cells from transplanted mice.

At 3 months, transplanted recipient spleen cells were harvested and B-cells sorted in identical fashion for previous *ex vivo* stimulations. GFP^+^mCherry^+^ B220^+^ cells represent engrafted (GFP^+^) and *Traf6* sgRNA-transduced (mCherry^+^) donor M146aKOCas reconstituted B-cells. As expected, B-cells transduced with negative control sgNT (non-targeting, red) showed similar increased cell counts as miR-146aKO splenic B-cells (**Figure 5C**). However, responses were decreased when *Traf6* was deleted using sgA (black) and sgB (gray). Hence, a modest reduction in *Traf6* (black) led to an attendant moderately decreased cell count response in miR-146aKO cells, whereas near-total *Traf6* deletion in B-cells (gray) led to a near-complete loss of expansion. TRAF6’s importance in B-cell responses *in vitro* to TLR ligands and activation of NFkB and MAPK pathways has been confirmed previously in non-miR regulated contexts (38). Conversely, engrafted GFP^+^ B-cells, which lack the targeting vector (non-*Traf6* deleted), behaved like miR-146KO cells, with preserved cellular responses, as measured by overall cell count, in response to both LPS and CpG (**Figure 5D**). In summary, by using the CRISPR/Cas9 gene editing system, we demonstrate that knockdown of downstream miR-146a target, *Traf6*, leads to a graded reversal of B-cell phenotype driven by miR-146a deficiency.

### Small molecule-based inhibition of TRAF6 results in decreased miR-146aKO B-cell proliferative response

**Figure 6:**
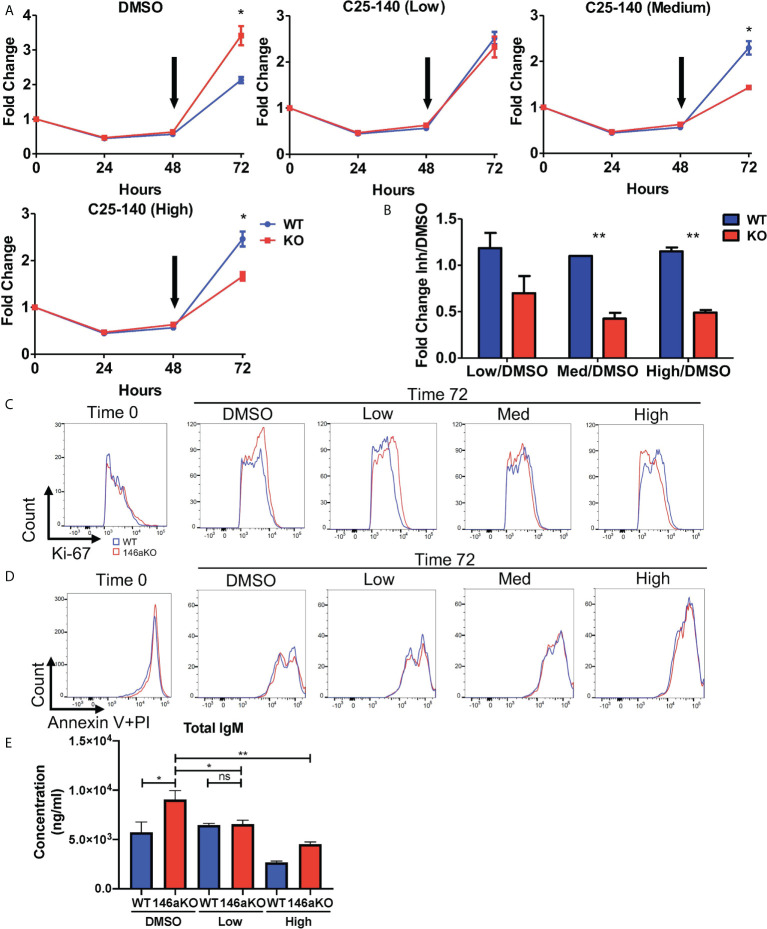
Pharmaceutical direct TRAF6 inhibition leads to graded decrease of miR-146a mediated B-cell proliferative response. **(A)** miR-146aKO splenic B-cells were stimulated with LPS, and TRAF6 inhibitor compound, C25-140 (5uM low dose; 15um medium dose; 30-40uM high dose), was added at 48 hours (black arrow) to assess the effects of cell count at 72 hours. Representative of 4-8 mice/group in duplicates, confirmed in three independent experiments. **(B)** Analysis of cellular response at 72 hours showed C25-140 inhibitor affecting KO cells greater than WT. All analysis used t test for respective time point comparisons. **(C)** Corresponding FACS analysis showed higher KO *vs.* WT Ki-67 proliferation at 72hrs in control group, which is reversed with dose dependent inhibitor addition. **(D)** Annexin V with propidium iodide staining showed no differences between WT and KO groups at 72hrs. **(E)** Supernatants from *in vitro* stimulation with TRAF6 inhibition at 72hr show a reduction in total IgM secretion in miR-146aKO B cells. *P < 0.05; **P < 0.01; ns, non-significant.

Given the reduction of TRAF6 leading to reversal of miR-146a’s B cell phenotype, we then sought to determine if pharmacologic inhibition of TRAF6 may similarly correct miR146a-deficient B cell phenotypes. A recently described small molecule inhibitor, C25-140, which targets the TRAF6-Ubc13 interaction, was shown to reduce NFkB activation in primary human PBMCs and murine T cells (17), although B cells were not described. Hence, we wanted to examine the effects of this TRAF6 inhibitor on B cells in our miR-146aKO model. Based on the published dose titrations on PBMCs and T cells, we added C25-140 at a low dose (5uM), medium dose (15uM), or high dose (30-40uM) to LPS stimulated B-cells at 48 hours and examined the effect of on B-cell proliferation at the 72 hour time point. While DMSO-treated miR-146a KO cell counts are higher than WT at 72hrs, addition of increasing doses of TRAF6 inhibitor reverses this trend so that WT B cells outgrow KO cells (**Figure 6A**) after 24 hours of culture with inhibitor. Indeed, at 72 hours, at all concentrations of the inhibitor, miR-146a KO B cells are more sensitive to C25-140 inhibition than WT (**Figure 6B**). In line with these observations, Ki-67 staining was initially higher in miR-146a KO than WT B cells in respective control (DMSO) groups but reversed with increasing doses of TRAF6 inhibitor (**Figure 6C**). There was no significant difference in Annexin V with propidium iodide staining at this time (**Figure 6D**). Furthermore, we previously showed that LPS-stimulated miR146aKO cells have higher IgM secretion than WT cells (**Figure 2G**). Here, we determined that C25-140 treatment led to a significant, dose-dependent reduction in secretion of IgM in the *in vitro* culture supernatants for LPS stimulated miR-146aKO B cells (**Figure 6E**). Interestingly, in contrast to miR-146aKO cells, WT changes in B-cell proliferation and IgM secretion were uncoupled at higher doses, which may represent differential dependency of these B-cell functions on TRAF6 or may be due to concentration and time of treatment. Future studies in assessing C25-140’s specific pharmokinetic effects will be useful.

Lastly, there was a significant decrease in expression of *Gbp9*, one of the downstream *Traf6* genes shown previously, with high dose C25-140 treatment (**Supplement 5C**). In summary, pharmacologic inhibition of TRAF6 with C25-140 led to decreased cellular proliferation in stimulated B cells, with a selective effect on miR-146a KO cells, again highlighting the importance of TRAF6 activation in driving the B-cell phenotype.

## Discussion

In summary, our work here demonstrates that miR-146a is induced following stimulation and regulates T-independent B-cell responses. MiR-146a KO mice show higher antigen specific Ig responses, with increased plasma cell subsets, in response to immunization. MiR-146aKO B-cells have heightened cellular responses and cell surface activation in *in vitro* assays using T-independent stimuli. This heightened response was particularly prominent in the marginal zone B-cell subset. Underlying these phenotypes, we found enrichment of the IFNγ pathway genes. We focused on known miR-146a target, *Traf6*, which has been described as a critical factor in mediating miR-146a function in myeloid and T-cells subsets. Using an endogenously developed MSCV-based CRISPR/Cas9 retroviral vector, we found that *in vivo* reduction of *Traf6* led to decreased proliferation of B-cells in miR-146aKO mice. Furthering this finding, we demonstrated that pharmacologic inhibition of TRAF6 led to a significant and selective inhibition of B-cell proliferation in miR-146a KO mice.

Previously published studies by us and others have shown that *miR-146a^-/-^* mice have exaggerated macrophage responses to LPS stimulation, heightened T follicular helper cells and germinal center B-cell responses, T-cell hyper-activation, and Treg dysregulation (2–7, 9). These are consequences of loss of feedback regulation *via* derepression of various miR-146a targets, including *Traf6, Irak1* and *Stat1* among others. In B-cells, previous work has examined the GC B-cell response, showing that B-cell specific deletion of miR-146a (“B-KO”) led to spontaneous and enhanced GC reactions associated with high affinity antibody production *via* CD40 pathway targets (9). However, our primary focus in this study was to assess the role of miR-146a in regulating ‘non-GC’ extrafollicular B-cell responses. While our study did not use conditionally targeted miR-146a deletion in B-cells, our use of T-independent stimuli, isolated *in vitro* stimulations, and concordant evidence from independent studies (9, 21, 39) support a B-cell intrinsic role for miR-146a. Hence, elucidating the lesser studied role of miR-146a regulation to T-independent stimuli may provide important insights into how B-cells respond in inflammatory contexts that may serve as initiators and/or propagators of autoimmune diseases.

Dysregulation of B-cells has been implicated in autoimmune diseases such as systemic lupus erythematosus (SLE), Sjogren’s disease, and dermatomyositis. These diseases show a complex pathogenesis, best demonstrated in SLE, involving abnormalities in innate immune cells, loss of T- and B-cell tolerance, autoantibody production, and abnormalities in the nuclear factor kappa B (NFkB) and interferon pathways (IFN) (40–42). Although it has long been established that germinal center (GC)-dependent memory B and long-lived plasma cells produce pathogenetic autoantibodies (43), more recent studies suggest that newly activated naïve B-cells without the need for affinity maturation may also contribute (44, 45). Indeed, recent work demonstrates the importance of extrafollicular (EF) generation of autoantibodies in humans, in which activated naïve B cells produce antibody secreting cells with significant autoreactivity, including anti-dsDNA (44). Interestingly, such unmutated, activated naïve B cells may underlie increases in anti-dsDNA titers during acute autoimmune disease flares. Our study simulates such T-independent stimulations in B-cells, showing a dependence on miR-146a to regulate such responses. In humans, we speculate that individuals with described genetic or acquired miR-146a deficiency (46, 47) may develop chronic TLR activation, immune overactivity, and autoimmunity, following what would usually be a self-limited infection with bacteria or virus. Prior work has suggested that infection with the Epstein-Barr virus (EBV) may represent one such initiator (40). Interestingly, EBV directly infects B-cells, and studies have shown that EBV proteins interfere with GC pathways, thus favoring EF responses in B-cells (48, 49). Hence, further interrogation of miRNAs in extrafollicular B-cell responses and the resolution of “culprit” infections may reveal novel pathogenetic insights into autoimmune disease.

At the molecular level, TLR binding to ligands activates recruitment of signaling molecules including TRAF6, IRAK1, and MyD88 to induce downstream inflammatory pathways, such as NFkB, MAPKinase, or IFN secretion, depending on the cell type and inflammatory context. Modulating levels *in vivo* of the known molecular target *Traf6* led to a cellular phenotypic reversal of the exaggerated *in vitro* responses. Hence, the B-cell proliferative phenotype that we observed was at least partially mediated by TRAF6 upregulation in the miR-146aKO mice. Regarding the importance of this pathway in regulating autoimmunity, our colleagues found reduction of inflammatory cytokines, reduced overall splenic cellularity, reduced spleen weight, and improvement in aberrant myeloproliferative phenotype in *miR-146a^-/-^Traf6^+/-^* mice, which manifested reduced levels of TRAF6 similar to WT (34). Linking this to human disease, two translational studies have reported an inverse correlation of miR-146a expression (i.e. low) and TRAF6 (i.e. high) in PBMCs and/or renal biopsies in SLE patients (50, 51). It is important to note that biologically, a single microRNA has many cell-specific gene targets in various molecular pathways that it can regulate in tandem to dynamically respond to inflammatory processes. Hence, although other gene targets of miR146a repression in B-cells exist, identifying key targets will allow for direct clinical translation into therapies. Here we show that in B-cells, *Traf6* is a major target of miR-146a and demonstrate proof-of-principle that pharmacologic TRAF6 modulation can impact the proliferation of miR-146a deficient B-cells.

TRAF6 is a key signal transduction molecule that connects TLR stimulation to downstream NFkB activation. In addition to its role in inflammatory pathways, deregulated TRAF6 expression appears to be a key feature in hematologic malignancies (52). In normal hematopoiesis, Traf6 is required for hematopoietic stem cell function (35), and is thought to be required for activation of B-cells *via* TLR signaling (38). TRAF6 is a E3 ubiquitin ligase, and this activity has recently been the focus of targeting efforts. Importantly, a TRAF6 small molecular inhibitor was recently described with activity in primary human and mouse cells, resulting in improved disease outcomes in rheumatoid arthritis and psoriasis mouse models (17). B-cell proliferation demonstrated a miR-146a-dependent sensitivity to chemical inhibition of TRAF6 with C25-140, the inhibitor described above. Finding further vulnerabilities in miR-146a KO B-cells will be enabled in future studies by combining C25-140 treatment with CRISPR libraries to explore other key targets in our B-cell activation system. Such key vulnerabilities may lie within the interferon pathway; for example, the finding of stronger induction of the IFNγ pathway in miR-146aKO B-cells indicates that B-cells lacking miR-146a may have sustained responsiveness and/or secretion of interferons. The genesis of interferon signaling in the miR-146a KO B-cells is not clear- the possibilities include *de novo* B-cell produced interferon, or it may represent cross-talk between TLR signaling pathways and IFN signaling. Indeed the former possibility was recently described in autoimmune disease, albeit as part of a T-cell dependent B-cell response (53). The latter possibility is slightly favored here, since the difference in interferon signaling was observed more strikingly after TLR stimulation of the B-cells. Nonetheless, combinatorial targeting of multiple miR-146a targets, including those in the interferon pathway, may provide novel avenues to treat autoimmune diseases in the future.

In summary, understanding the functions of microRNAs in specific cellular contexts and their molecular targets provide important insights into how epigenetic regulation of immune responses work not only in individual cells, but also as interrelated responses to inflammatory processes. MiRs have often been considered ‘fine tuners’ of the immune system in biologically dynamic states, which makes their utilization in assessing *in vivo* disease activity levels compelling. This has implications in cell specific miR (i.e. miR-146a-mimic conjugated to CpG) (54) and molecular gene target applications (TRAF6 inhibitors, etc) for future therapeutic application in autoimmune diseases, that are currently being explored. Hence, our study sheds light on a previously the understudied role of miR-146a’s regulation of B-cell in extrafollicular T-independent responses, their potential role in autoimmunity, and possible future clinical applications.

## Data availability statement

The data presented in the study are deposited in the NCBI SRA, accession number PRJNA723980, https://www.ncbi.nlm.nih.gov/bioproject/PRJNA723980.

## Ethics statement

The animal study was reviewed and approved by UCLA Office of Animal Research Oversight.

## Author contributions

JK and DR contributed to the conception, design, and written manuscript. JK, TT, MP, YY, AJ, C-HT, and KR contributed to experiments. JK, TT, and MP contributed to analysis and sections of writing manuscript. DC performed RNA-Seq analysis. All authors contributed to manuscript revision, read, and approved the submitted version.

## Funding

Supported by NIH National Institute of Arthritis and Musculoskeletal and Skin Diseases (K08AR072787), NIH National Cancer Institute (R01CA166540; R01CA264986), NIH NIAID (R21AI132869). Flow cytometry was performed in the Eli and Edythe Broad Center of Regenerative Medicine and Stem Cell Research UCLA Flow Cytometry Core Resource and the UCLA JCCC/CFAR Flow Cytometry Core Facility that is supported by NIH AI-28697, P30CA016042, the JCCC, the UCLA AIDS Institute, and the David Geffen School of Medicine at UCLA.

## Conflict of interest

The authors declare that the research was conducted in the absence of any commercial or financial relationships that could be construed as a potential conflict of interest.

## Publisher’s note

All claims expressed in this article are solely those of the authors and do not necessarily represent those of their affiliated organizations, or those of the publisher, the editors and the reviewers. Any product that may be evaluated in this article, or claim that may be made by its manufacturer, is not guaranteed or endorsed by the publisher.
