# Supplementary material for: Regulation of T-independent B-cell responses by microRNA-146a
Source: Front Immunol. 2022 Sep 12;13:984302. doi: 10.3389/fimmu.2022.984302 (PMC9511149; doi:10.3389/fimmu.2022.984302)
Supplement: Supplementary file 1 [file DataSheet_1.pdf]

Regulation of T-Independent B-Cell Responses by MicroRNA-146a

King et al…

Supplement

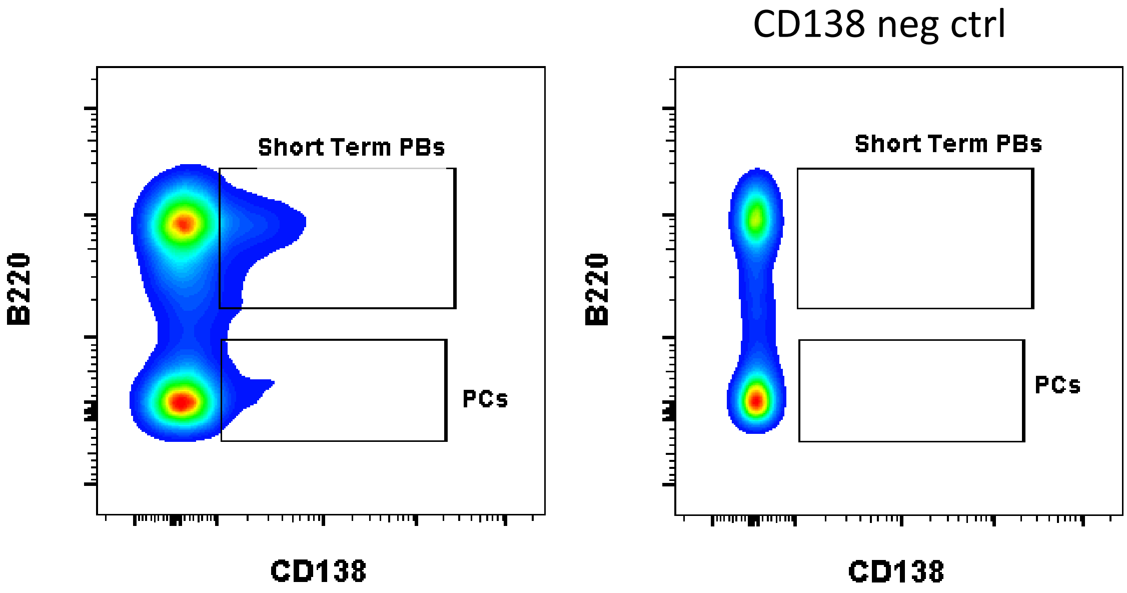

Supplement 1: Representative FACS plot of splenic plasma cell subsets from primary immunization response to T-independent antigen (left) vs. B220+CD138- negative staining control (right).

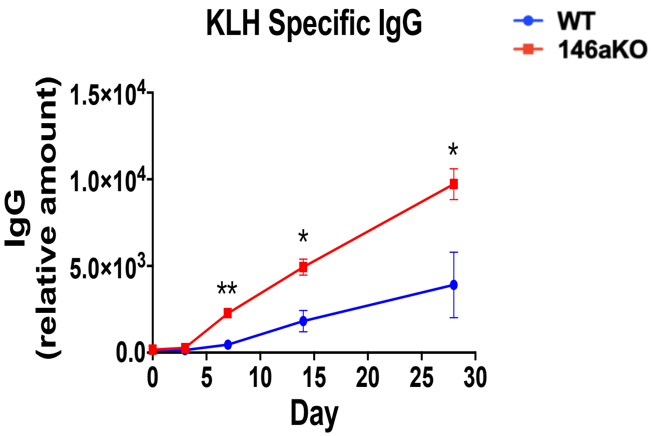

Supplement 2A: miR-146aKO B-cells have higher peptide specific IgG secretion in response to T-dependent antigen than WT. After *in vivo* immunization with T-dependent antigen Keyhole Limpet Hemocyanin (TNP-KLH), KLH specific IgG responses were higher in miR-146aKO than in WT (*t* test; **P* ≤ 0.05, ***P* < 0.01). n = 3 mice/group. Data represent mean ± SEM.

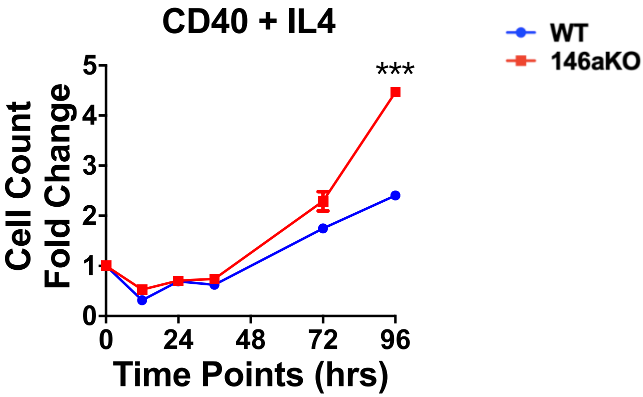

Supplement 2B: MiR-146aKO splenic B-cells activated with T-dependent stimuli have higher cell counts than WT. B-cells were stimulated with anti-CD40 (5ug/ml) along with IL-4 (5ng/ml). Representative of 3 mice/group in duplicates or triplicates, confirmed in 2 independent experiments. (*t* test; ****P* < 0.001).

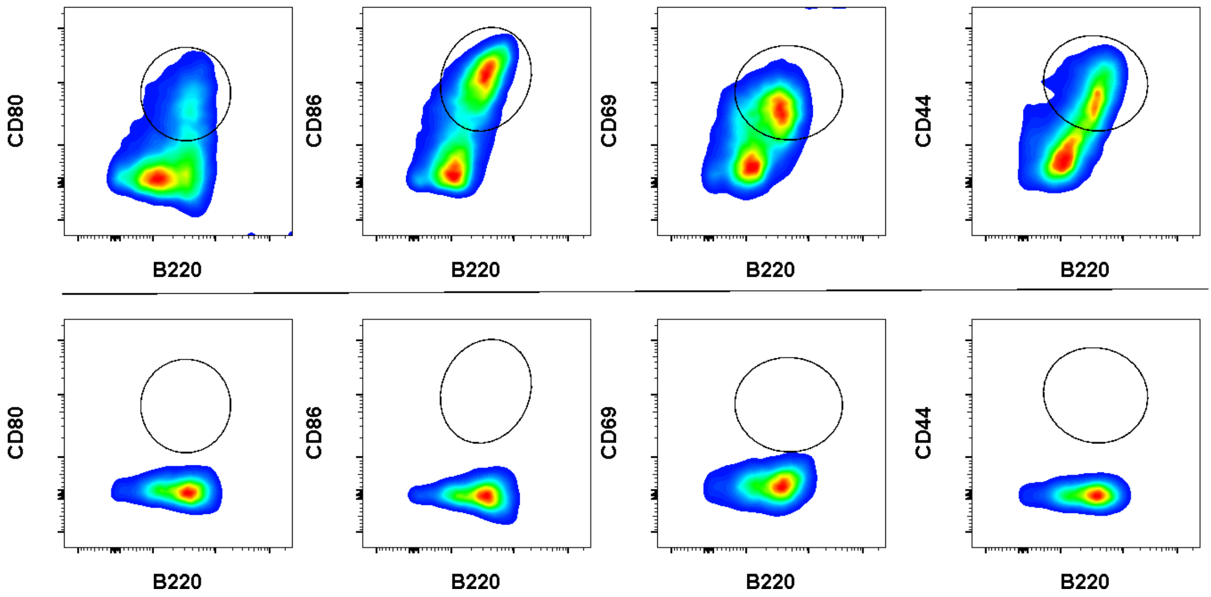

Supplement 3. Representative FACS plots for splenic B220+ cell surface activation markers (top panel) with FMOs (bottom panel).

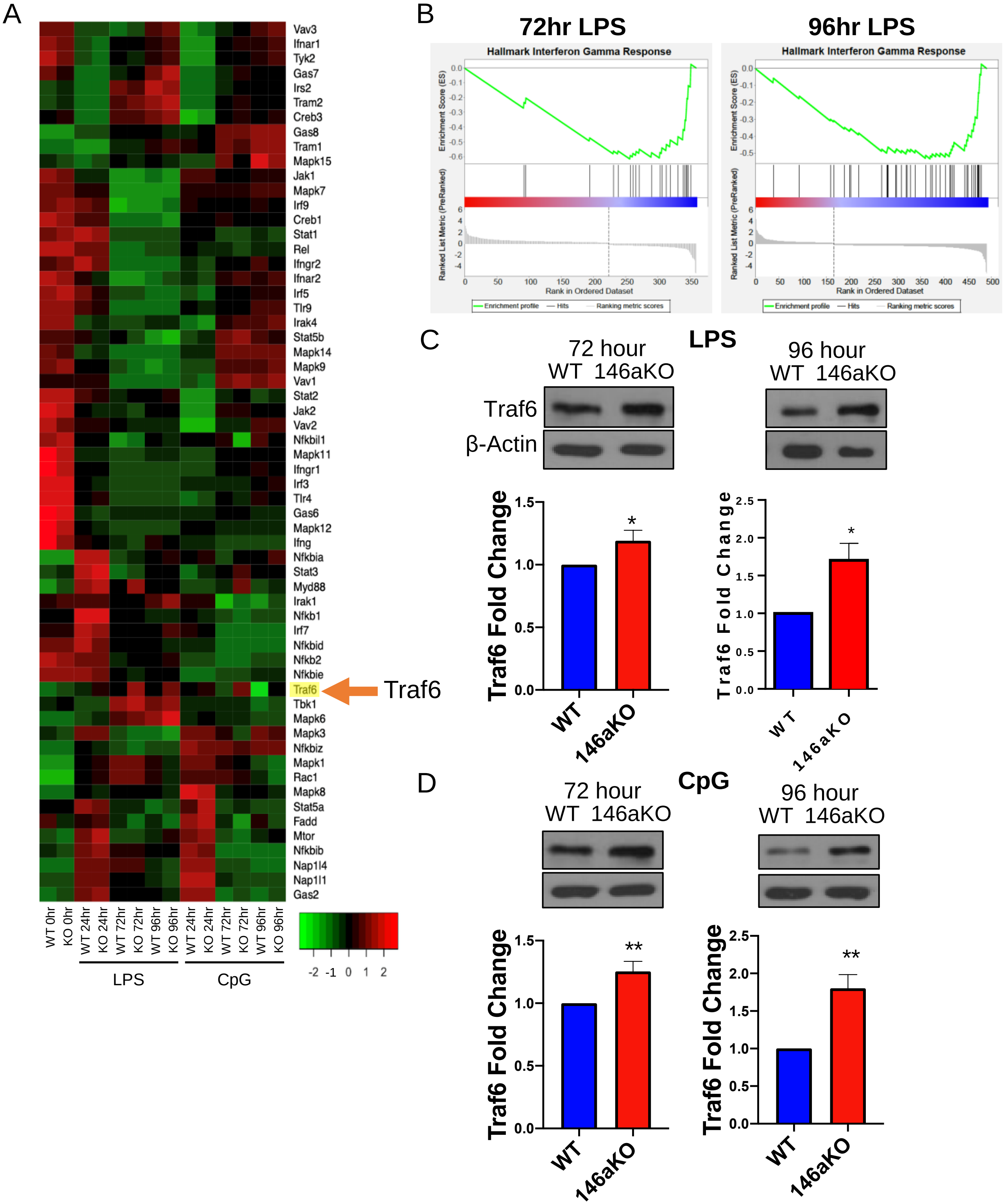
A
Vav3
Ifnar1
Tyk2
Gas7
Irs2
Tram2
Creb3
Gas8
Tram1
Mapk15
Jak1
Mapk7
Irf9
Creb1
Stat1
Rel
Ifngr2
Ifnar2
Irf5
Tlr9
Irak4
Stat5b
Mapk14
Mapk9
Vav1
Stat2
Jak2
Vav2
Nfkbil1
Mapk11
Ifngr1
Irf3
Tlr4
Gas6
Mapk12
Ifng
Nfkbia
Stat3
Myd88
Irak1
Nfkb1
Irf7
Nfkbid
Nfkb2
Nfkbie
Traf6
Tbk1
Mapk6
Mapk3
Nfkbiz
Mapk1
Rac1
Mapk8
Stat5a
Fadd
Mtor
Nfkbib
Nap1l4
Nap1l1
Gas2
Traf6
WT 0hr
KO 0hr
WT 24hr
KO 24hr
WT 72hr
KO 72hr
WT 96hr
KO 96hr
WT 24hr
KO 24hr
WT 72hr
KO 72hr
WT 96hr
KO 96hr
LPS
CpG
-2 -1 0 1 2
B
72hr LPS
Hallmark Interferon Gamma Response
Enrichment Score (ES)
Ranked List Metric (PreRanked)
Rank in Ordered Dataset
Enrichment profile
Hits
Ranking metric scores
96hr LPS
Hallmark Interferon Gamma Response
C
LPS
72 hour
WT 146aKO
96 hour
WT 146aKO
Traf6
β-Actin
Traf6 Fold Change
WT
146aKO
*
D
CpG
72 hour
WT 146aKO
96 hour
WT 146aKO
Traf6 Fold Change
WT
146aKO
**

Supplement 4: *Traf6* expression increases with T-independent antigen stimulation of B-cells. (**A**) Heatmap from the RNA-Seq data showing differentially expressed IFN targeted genes. miR-146a molecular target, *Traf6*, is highlighted in yellow. (**B**) Gene set enrichment analysis (GSEA) of genes differentially expressed between WT and miR-146aKO activated B-cells shows enrichment in Interferon γ pathways. 72hrs Normalized Enrichment Score: -1.96; FDR q value: 0.0. 96hrs Normalized Enrichment Score: -1.85; FDR q value: 0.046. **(C)** Protein expression via Western Blot of TRAF6 in B-cells when stimulated with LPS and (**D**) CpG. t test; $*P < 0.05$, $**P < 0.01$. Data represent mean ± SEM. Graphs combine 3 independent experiments (n = 3-4 mice/group/exp).

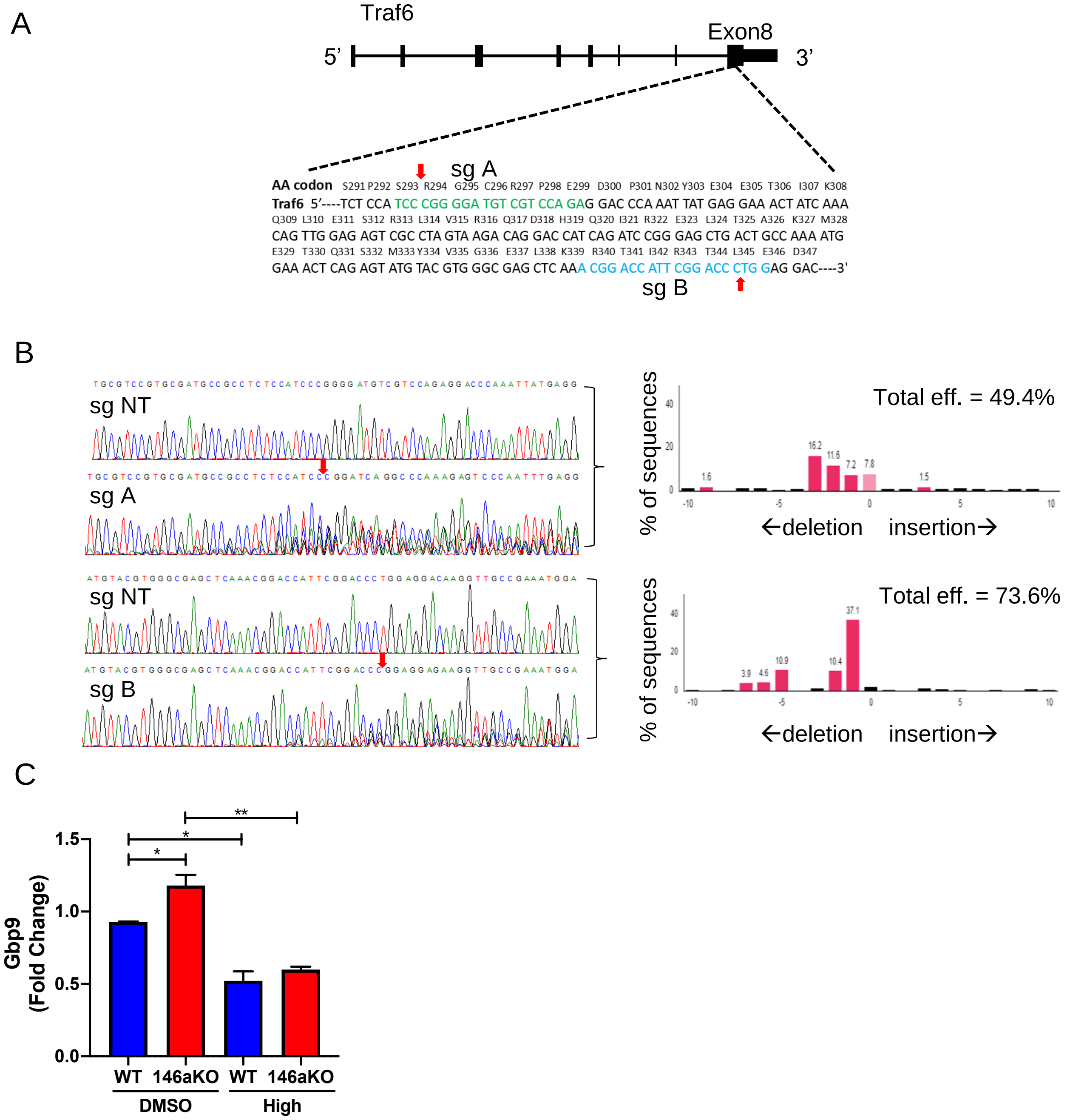

Supplement 5: Traf6 sgRNAs for CRISPR/Cas9. (**A**) sgRNAs (sg A and B) location on Exon 8 of *Traf6*. AA codon sequence indicates the detail location. (**B**) TIDE analysis of engrafted primary bone marrow cells transduced with sg NT, A, and B (GFP+ mCherry+ cells) respectively. Left panel is the sequence, red arrows show the position of double strand breaks (DSBs) that should be introduced by CRISPR/Cas9; right panel is the TIDE results, total eff. means sum for all the types of gene editing. **(C)** RT-qPCR of downstream *Traf6* gene, *Gbp9*, in LPS-stimulated B-cells treated with DMSO or high dose of C25-140. t test; *$P < 0.05$, **$P < 0.01$.

Supplementary Table 1. List of antibody stains used in FACS analysis.

Antigen	Clone
B220	RA3-6B2
CD21	7E9
CD23	B3B4
CD80	16-10A1
CD86	GL-1
CD69	H1.2F3
CD44	IM7
CD40	HM40-3
CD138	281-2
CD93	AA4.1
GL7	GL7
CD95	SA367H8
IgD	11-26c.2a
CD3e	145-2C11
CD11b	M1/70
Ly-6c	HK1.4

Supplementary Table 2. sgRNA and primer sequences used in this study.

sgRNA	Sequence
sg A (F)	TTTGTCTGGACGACATCCCCGGGA
sg A (R)	AAACTCCCGGGGATGTCGTCCAGA
sg B (F)	TTTGACGGACCATTCGGACCCTGG
sg B (R)	AAACCCAGGGTCCGAATGGTCCGT
sg NT (F)	TTTGCGAGGTATTCGGCTCCGCG
sg NT (R)	AAACCGCGGAGCCGAATACCTCG
m-Traf6-F	GCACAAGTGCCCAGTTGAC
m-Traf6-R	TGCAAAATTGTCGGGAAACAGT
m-Cd72-F	CATACCCTCGGAAGTCTGGA
m-Cd72-R	GCCTCCACTTCTTGCTCATC
m-Ccng1-F	TAAGTGGCCGAGATTTGACC
m-Ccng1-R	ATGGTCTCAGGAATCGTTGG
m-Gbp9-F	TGTGCAGTCTCAGACCAAGG
m-Gbp9-R	AAGCACACTTAGGGCGAAGA
